# Nanoparticles Based on Quaternary Ammonium Chitosan-methyl-β-cyclodextrin Conjugate for the Neuropeptide Dalargin Delivery to the Central Nervous System: An In Vitro Study

**DOI:** 10.3390/pharmaceutics13010005

**Published:** 2020-12-22

**Authors:** Chiara Migone, Letizia Mattii, Martina Giannasi, Stefania Moscato, Andrea Cesari, Ylenia Zambito, Anna Maria Piras

**Affiliations:** 1 Department of Pharmacy, University of Pisa, Via Bonanno 33, 56126 Pisa, Italy; chiara.migone@for.unipi.it (C.M.); martinagiannasi1990@gmail.com (M.G.); anna.piras@unipi.it (A.M.P.); 2 Department of Clinical and Experimental Medicine, University of Pisa, 56126 Pisa, Italy; letizia.mattii@med.unipi.it (L.M.); stefania.moscato@unipi.it (S.M.); 3 Interdepartmental Research Centre “Nutraceuticals and Food for Health”, University of Pisa, 56100 Pisa, Italy; 4 Department of Chemistry and Industrial Chemistry, INSTM—University of Pisa Research Unit (UdR Pisa), University of Pisa, Via Moruzzi 13, 56124 Pisa, Italy; andrea.cesari@dcci.unipi.it

**Keywords:** mucoadhesive nanoparticles, Caco-2 monolayer permeation, bEnd.3 monolayer permeation, blood–brain barrier permeation, peptide brain targeting

## Abstract

Peptide oral administration is a hard goal to reach, especially if the brain is the target site. The purpose of the present study was to set up a vehicle apt to promote oral absorption of the neuropeptide dalargin (DAL), allowing it to cross the intestinal mucosal barrier, resist enzymatic degradation, and transport drugs to the brain after crossing the blood–brain barrier. Therefore, a chitosan quaternary ammonium derivative was synthesized and conjugated with methyl-β-cyclodextrin to prepare DAL-medicated nanoparticles (DAL-NP). DAL-NP particle size was 227.7 nm, zeta potential +8.60 mV, encapsulation efficiency 89%. DAL-NP protected DAL from degradation by chymotrypsin or pancreatin and tripled DAL degradation time compared to non-encapsulated DAL. Use of DAL-NP was safe for either Caco-2 or bEnd.3 cells, with the latter selected as a blood–brain barrier model. DAL-NP could also cross either the Caco-2 or bEnd.3 monolayer by the transepithelial route. The results suggest a potential DAL-NP ability to transport to the brain a DAL dose fraction administered orally, although in vivo experiments will be needed to confirm the present data obtained in vitro.

## 1. Introduction

As it is now well known, the administration of drugs, especially of the peptide nature, to the central nervous system by the oral route, is extremely problematic [1,2]. Indeed, the blood–brain barrier (BBB) allows access to the central organ to only a few molecules, and the access is limited to low-molecular-weight lipid molecules (MW < 400 Da), via passive diffusion, and to specific endogenous ligands, via mediated transport. Furthermore, the lipophilicity of lipophilic drugs of MW < 400 Da is a key factor for permeation. In fact, the ideal octanol–water partition coefficient at pH 7.4 for transport through the BBB is between 1 and 10, corresponding to a log P from 0 to 1 [3]. It should be considered that the BBB also uses specific defense mechanisms (ATP-binding cassette transporters, ABC) that cause efflux of internalized molecules. These are poured back into the blood stream, and are therefore prevented from crossing the barrier [4].

The use of nanoparticulate vehicles has opened new possibilities of transport, of either the passive or active type, across the BBB, and is currently one of the more studied strategies for drug targeting to the brain [5,6]. However, for the therapeutic administration of pharmacologically active peptides, resorting to the parenteral route is necessary. In fact, the oral administration of peptides for systemic action is rare, as it is limited by the poor intestinal absorption of these drug types. This is due to both a prompt degradation in the intestinal lumen by an enzyme-rich digestion system and a poor permeability across the mucosal intestinal barrier.

The purpose of the present work was to study a vehicle able to promote the oral absorption of dalargin (DAL) by resisting enzymatic degradation, thus helping the drug across the mucosal intestinal barrier, and transporting it across the BBB. DAL is a neuropeptide belonging to the enkephalin family, possibly pharmacologically acting on the central nervous system. In the Russian Federation, it is commercialized as a medicinal injectable solution (Dalargin), with therapeutic applications in the treatment of gastric ulcer, arrhythmias, and alcohol withdrawal syndrome. DAL has a potentially powerful antinociceptive effect that cannot be exploited since it is rapidly metabolized upon injection and it is not able to permeate the BBB [7,8]. Presently, its brain action is believed to be in fact due to an active metabolite [9].

The carrier focused on for this study is of the nanoparticulate type, thanks, in particular, to the nanoparticle ability to transport a comparatively high active principle amount, and to the increased analgesic effect observed in mice by loading DAL into synthetic poly(butylcyanoacrylate) nanoparticulate carriers [10]. In this context, chitosan-based nanoparticles have shown excellent control over peptide control release [11], and recent studies have shown manifold properties of chitosan quaternary ammonium derivatives (N^+^-rCh) conjugated with methyl-β-cyclodextrin (MCD) [12]. These have been exploited to promote the oral absorption of hydrophobic small molecules [13,14]. Moreover, the DAL stabilizing power by the conjugate quaternary ammonium chitosan–MCD (N^+^-rCh-CD) in the presence of digestive enzymes has been demonstrated [15]. In fact, in the case of peptide active ingredients, cyclodextrins are often studied because the inclusion of hydrophobic amino acid residues in the toroid determines a stabilization of the tertiary structure and the slowdown of enzymatic reactions toward the active ingredients [16]. However, although N^+^-rCh-CD was shown to be able to protect DAL from enzymatic degradation, the DAL/N^+^-rCh-CD complex was not shown to be able to promote intestinal absorption of the peptide.

Therefore, in the present study, the N^+^-rCh-CD conjugate, which had shown an ability to protect DAL from enzyme digestion, was used to prepare DAL-encapsulating nanoparticles (DAL-NP) intended for oral administration. The DAL-NP ability to promote DAL transport through the Caco-2 cell monolayer, simulating the intestinal epithelium, or the bEnd.3 cell monolayer, chosen as the BBB model, were investigated.

## 2. Materials and Methods

### 2.1. Materials

Sodium nitrite, 2-diethylaminoethyl chloride hydrochloride (DEAE-Cl∙HCl), α-chymotrypsin (CHT), pancreatin (PAC), dimethyl sulfoxide (DMSO), fluorescein isothiocyanate-dextran (FITC), 1,6-hexamethylene diisocyanate (HMDI), triethylamine (TEA), sodium triphosphate pentabasic (TPP), bicinchoninic acid assay (BCA), all salts for the preparation of buffers, tripolyphosphate, trehalose dihydrate, and Sephadex G-15 (St. Louis, MO, USA) were purchased from Merk. Chitosan was purchased from Faravelli (Milan, Italy; molecular weight = 300 kDa). Dalargin ((D-Ala2)-Leu Enkephalin-Arg) was purchased from Bachem (Weil am Rhein, Germany). 2-Methyl-β-cyclodextrin (MCD; DS = 0.5) was purchased from Roquette (Lestrem, France). Standard pullulans (642 ÷ 6.10 kg/mol) were purchased from American Polymers Standard Corporation (8680 Tyler Blvd., Mentor, OH, USA), and dialysis membranes (MWCO 12500) were purchased from Spectra/Por (Sigma, Los Angeles, CA, USA). The deuterated solvents heavy water (D_2_O) and Sodium deuteroxide (NaOD) (30%) were purchased from Deutero GmbH (Kastellaun, Germany).

HMDI and DMSO were distilled under reduced pressure (65 ℃/0.2 mbar and 24 ℃/0.2 mbar, respectively); TEA was refluxed on calcium hydride and distilled before use. MCD and DEAE-Cl∙HCl were dried under vacuum at 37 ℃ before use.

The Caco-2 and bEnd.3 cell lines were purchased from the American Type Culture Collection LGC standards ((ATCC HTB-37), Milan, Italy) and propagated as indicated by the supplier. The minimum essential medium (MEM), non-essential amino acid, 0.01 M pH 7.4 Dulbecco’s phosphate-buffered saline (DPBS), phosphate buffered-saline free of calcium and magnesium (PBSA), fetal bovine serum (FBS), glutamine, antibiotics (penicillin/streptomycin), and Hank’s balanced solution were purchased from Sigma (Milan, Italy). Antimycotic was supplied from Invivogen (San Diego, CA, USA). Cell proliferation reagent (WST-1) was provided by Roche diagnostic (Milan, Italy).

### 2.2. Synthesis and Characterization of N^+^-rCh-CD

Commercial chitosan depolymerization was carried out as described by Mao [17]. The depolymerized product was subsequently quaternized and conjugated with MCD to obtain the N^+^-rCh-CD derivative, as already described previously [15,18]. N^+^-rCh-CD was labeled with FITC as previously described [14]. To characterize the N^+^-rCh-CD derivative, we made ^1^H NMR measurements by the Bruker Advance II spectrometer, working at 400 MHz, at the temperature of 25 ± 0.1 ℃, on samples dissolved in D_2_O at the concentration of 1% *w*/*v*. Then, the deacetylation degree; the quaternization degree, in terms of substitution per repeating unit; the length of the pendant chain; and the conjugation degree of cyclodextrin with the quaternized polymer were calculated as previously described [15].

The determination of N^+^-rCh-CD MW was carried out by determining the variation of the scattering intensity [19]. For this purpose, an aqueous (Milli-Q) N^+^-rCh-CD stock solution (3 mg/mL) was prepared and used to obtain samples in the 0.15–1.50 mg/mL concentration range. These were filtered (0.22 µm) and analyzed in quartz cuvettes at the analysis temperature of 25 ℃ by dynamic light scattering technique (Malvern Zetasizer Nano ZS, Malvern Panalytical Ltd, Malvern, United Kingdom). Toluene was used as a reference solvent and the differential increment value of refraction index as a function of concentration was set at 0.192 mL/g [20]. The accurate values of solution concentrations were determined gravimetrically after removing the solvent by lyophilization (VirTis AdVantage wizard 2.0, SP Scientific). For lyophilization, the solutions were placed in Eppendorf tubes, congealed at −40 ℃, and sublimated at 30–40 mTorr pressure, with end point at 16 ℃.

### 2.3. Characterization of DAL Inclusion Complex

The DAL/N^+^-rCh-CD complex was characterized for stoichiometry (Job’s plot) and association constant (Benesi–Hildebrand fluorescence method), as previously described [15].

### 2.4. Preparation and Characterization of N^+^-rCh-CD-Based, DAL-Loaded NP

DAL-medicated nanoparticles (DAL-NP) were prepared by the ionotropic crosslinking of N^+^-rCh-CD with TPP. A 2.0 mg/mL N^+^-rCh-CD solution was obtained by dissolving the polymer in filtered deionized water under magnetic stirring overnight. The TPP (0.150 mg/mL) and DAL (1.0 mg/mL) solutions were prepared in filtered (0.22 µm) deionized water. The nanoparticle dispersion was formed spontaneously by mixing 0.250 mL of the polymer solution with 0.100 mL of the DAL solution and 0.150 mL of deionized, filtered water, then dropwise adding 0.500 mL of the TPP solution under stirring at room temperature.

For the biological permeation tests DAL-NP were prepared using an FITC-labeled polymer (DAL-NP-FITC). The formulations were prepared according to the above-described procedure but for the use of a labeled polymer.

The DAL-NP were purified by centrifugation (20 min, 8000 rpm at 4 ℃). The pellets were re-suspended and used for the morphological analysis, while the supernatants were used to evaluate the DAL loading ability of DAL-NP by the following indirect way. The free peptide concentration in the supernatants from the DAL-NP purification was determined by the bicinchoninic acid assay (BCA). The mix under test, containing 100 µL of BCA reactant and 100 µL of supernatant, was analyzed at 562 nm by the UV–VIS spectrophotometer Perkin Elmer Lambda-25. The encapsulation efficiency (EE) was calculated as the DAL fraction encapsulated with respect to the total amount used in the preparation. The loading ability (L%) was calculated as the weight ratio between amount of loaded drug and dried DAL-NP weight.

The products formulated in aqueous solution were lyophilized in glass bottles (−40 ℃, 40 mTorr, end point at 16 ℃). Before lyophilization, 9.5% trehalose was added as a cryoprotectant.

The nanoparticle size and zeta potential were determined in triplicate in 0.4 M phosphate buffer (PB pH 6.8) at 25 ℃ (Malvern Zetasizer Nano ZS). The analysis was performed on the re-suspended lyophilizate.

The nanoparticle morphology was assessed by scanning transmission electron microscopy (STEM) analysis (FEI Quanta 450 ESEM FEG) on nanoparticles purified by centrifugation, re-suspended in deionized water (1 mL), diluted 1:100 with ethanol, and placed on graphene supports. The STEM analysis was performed after solvent evaporation at room temperature (24 h).

### 2.5. DAL Release from DAL-NP

The lyophilized DAL-NP were suspended in 3.0 mL phosphate-buffered saline (PBS; pH 7.4) so as to obtain a DAL concentration of 126.7 µg/mL. The DAL-NP dispersion was kept 6 h at 37 ℃ in shaker water bath. At times 0, 1, 3, and 6 h, we withdrew 300 µL samples from the dispersion. These were centrifuged (15,000 rpm, 20 min, room temperature), and the supernatants were analyzed for DAL by the BCA test.

### 2.6. Assessment of the Enzymatic Degradation Kinetics

Stock 1 mg/mL solutions of DAL-NP or free DAL in filtered 0.4 M PB were used for the enzymatic digestion tests. The solutions were mixed with 0.36 mg/mL CHT or 0.18 mg/mL PAC to obtain solutions with a final DAL concentration of 0.50 mg/mL. The DAL-NP and free DAL samples were subjected to digestion in the presence of CHT or PAC at 37 ℃ in a shaker water bath (80 rpm). At pre-established intervals, samples were withdrawn, appropriately diluted with the mobile phase (4 ℃), and analyzed in triplicate for DAL by HPLC, as described previously [15]. The analysis was repeated in triplicate.

### 2.7. Cell Studies

#### 2.7.1. Cytotoxicity Studies of Caco-2 and bEnd.3 by the Tetrazolium Salt Test (WST-1)

To study the cytotoxicity of the DAL/N^+^-Ch-CD complex, we mixed 400 µL of polymer solution (3.175 mg/mL) and 380 µL of 1 mg/mL aqueous DAL, and the mix was lyophilized under the conditions described above. The DAL-NP were lyophilized and the lyophilized product was re-suspended in 250 µL of 0.22 µm filtered water. An aliquot of dispersion (200 µL) was withdrawn and added to 20 µL of Hank’s buffer solution 10× (HBSS 37 ℃, pH 6.8), and the mix was made to 2 mL with HBSS 1X as to have a DAL content of 380 µg/mL. The Caco-2 cells were subjected to DAL concentrations in the 17–150 µg/mL range, while the bEnd.3 cells were brought into contact with DAL concentrations in the 17–100 µg/mL range; more specifically, DAL was free in solution, contained in the DAL/N^+^-rCh-CD (1:1) complex, or encapsulated in NP. The cytotoxicity of the compounds under study was evaluated by the WST-1 assay. Caco-2 and bEnd.3 cells were seeded in each well of 96-well plates at a seeding density of 2 × 10^4^ cells per well, and left to proliferate for 24 h at 37 ℃ in 5% CO_2_. The culture medium was then removed and replaced with samples of DAL, DAL/N^+^-Ch-CD complex, and DAL-NP, as previously described. Afterwards, formazan dye absorbance was quantified at 450 nm with the reference wavelength of 655 by using an Enspire 230 (Perkin-Elmer, Waltham, MA, USA) multilabel reader.

#### 2.7.2. Studies of Permeation across Caco-2 Monolayer

The permeation studies were performed as described previously [15]. Briefly, a Caco-2 monolayer was obtained by cell seeding (10^5^ cells per well) on Transwell 12-well plates (pore size 0.4 m, area 1.12 cm^2^; Sigma-Aldrich, Saint Louis, MO, USA). Thereafter, 0.5 and 1.5 mL of culture medium were added to apical and basolateral compartments of the inserts, respectively; the culture medium was replaced every 2 days for 24 days. The transepithelial electrical resistance (TEER, Ω cm^2^) measurement during the permeatstudies was carried out using a voltmeter with rod electrodes (Voltmeter Millicells-ERS, Millipore, Molsheim, France). The TEER values for the Caco-2 cell monolayer grown on the Transwell filter were ion taken at time 0, every hour for 3 h, and at 24 h.

The samples tested were DAL and DAL-NP-FITC, with an initial DAL apical concentration of 150 µg/mL. Both the NP vehicle and the DAL were followed in the mucosal and basolateral phases. At 1 h intervals up to 3 h, the runs were interrupted and 500 µL was collected from the apical chamber and 1.5 mL from the basolateral chamber. The withdrawn samples were kept in ice and analyzed by HPLC. The evaluation of DAL contained in NP-FITC by HPLC implied subtracting the contribution of the fluorescein-labeled polymer from the peptide peak area. Therefore, with each sample to be analyzed, a specific fluorescence intensity was recorded by a spectrofluorometer from which the fluorescent polymer concentration to be injected in the HPLC apparatus could be extrapolated. The integrated area obtained from such an injection was subtracted from the total area obtained with the sample injected, thus obtaining the actual contribution from DAL to the total area.

#### 2.7.3. Permeation Studies across bEnd.3 Monolayer

The bEnd.3 cells, at a concentration of 5 × 10^4^ cells per well, were seeded on 12-well Transwell plates with polyester filters with 0.4 µm membrane pores and incubated 10 days at 37 ℃ in a 5% CO_2_ atmosphere. One and a half milliliters of full medium was added to the basal chamber, while a volume of 0.5 mL, including the cells, was added to the apical chamber. The medium was changed every 2 days. After 6 days culture, the medium of the basolateral chamber was replaced by serum-free medium and the cells were cultured for 4 more days before the permeation experiment. To evaluate the confluence of the cell monolayer, and during the permeation studies, we measured the TEER at time 0 and at each hour for 3 h, and then at 24 h, as previously described. The samples tested were DAL and DAL-NP-FITC, with an initial DAL apical concentration of 25 µg/mL. At 1 h intervals up to 3 h, the runs were interrupted and 500 µL was collected from the apical chamber and 1.5 mL from the basolateral chamber. Permeation of DAL and FITC-labeled vehicle into the basolateral chamber as well as DAL and FITC-labeled vehicle concentrations in the apical chamber were measured as described above.

## 3. Results

### 3.1. Characterization of N^+^-rCh-CD

The synthesis of N^+^-rCh and its conjugation with MCD were carried out according to a protocol developed in a previous study [14]. The polymer obtained had an 8.8% acetylation degree and a 42% degree of substitution of the quaternary chain with a N^+^/N ratio of 2 and 22% of grafted cyclodextrin. The studies of polymer characterization by the qEstimate software, using a cyclodextrin sample as an external standard, allowed quantification of the cyclodextrin content. The analysis confirmed a high grafting degree, corresponding to a cyclodextrin content of 52% *w*/*w*.

The weight average molecular mass of the conjugate as determined by the scattering intensity variation method was 222 ± 72 kg/mol, while the stoichiometry of the complex as determined by the Job’s plot method was 1:1. The tests were carried out at times 0, 1 h, and 24 h from preparation of samples, without finding any significant differences, as shown in Figures S1 and S2.

### 3.2. Characterization of DAL Inclusion Complexes

The formation of the DAL/MCD complex was studied and compared with that of the DAL-N*^+^*-rCh-CD complex and the physical N^+^-rCh/MCD mix. In all cases, the ligand mole fraction was 0.5, corresponding to a 1:1 DAL/MCD stoichiometry of complex.

The association constant (K_a_) values for the complexes DAL/MCD and DAL/N^+^-rCh-MCD were 126 ± 10 M^−1^ and 403 ± 37 M^−1^, respectively.

### 3.3. Characterization of DAL-NP

The physico-chemical characterization of DAL-NP was performed in terms of size distribution, zeta potential in physiological conditions, and morphology. These properties are of a fundamental importance in determining the stability and in vivo behavior of colloidal systems, as they influence the biodistribution, toxicity, drug release kinetics, and particle targeting ability [21].

**Figure 1:**
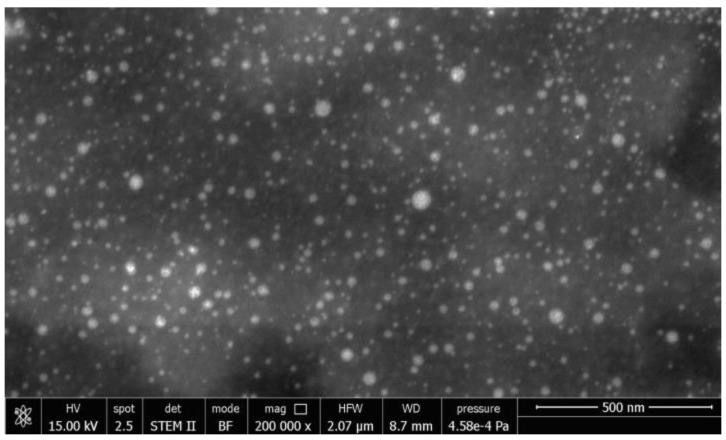
STEM micrography (200,000× *g* magnification) of dalargin (DAL)-medicated nanoparticles (DAL-NP), as purified by centrifugation.

A 227.7 nm mean DAL-NP diameter resulted with a polydispersity index of 0.238 ± 0.034, which can be considered typical of a homogeneous suspension. Moreover, the granulometric distribution curve (not reported) evidenced a one-size population of particles. The nanoparticle ζ potential was +8.60 mV in PB 0.4 M at pH 6.8. The morphological analysis carried out by STEM, found in Figure 1, confirmed the spherical shape and size homogeneity of particles.

The drug loading (L%) and encapsulation efficiency (EE%) values were 22.4 ± 0.2% and 89.1 ± 2.3%, respectively.

### 3.4. DAL Release from DAL-NP

**Figure 2:**
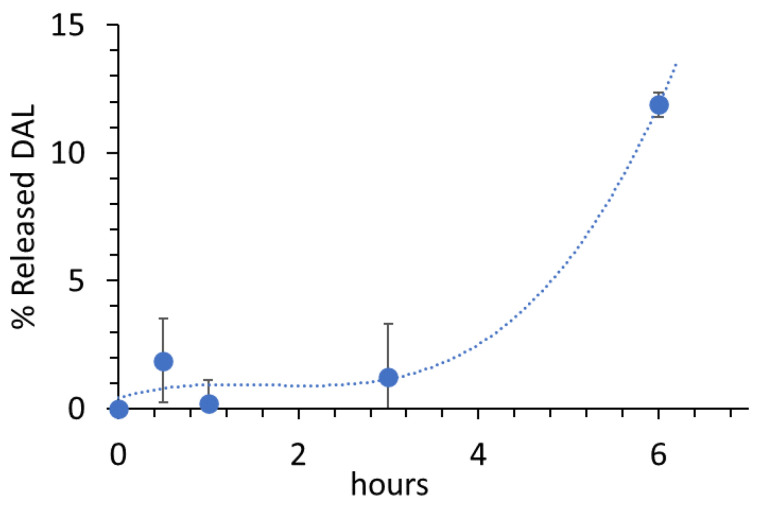
Percentage of DAL released from DAL-NP vs. time in phosphate-buffered saline (PBS). Data are the means ± standard deviation (SD) of two independent experiments.

Figure 2 shows DAL release data from DAL-NP. The release was monitored for a time compatible with the time needed for the nanosystems to be absorbed and reach the central nervous system [22]. It is interesting to note that the release was practically null for the first 3 h and underwent an abrupt increase after 6 h. Nevertheless, considering that only 10% of the dose was released in a 6 h time, it can be thought that the DAL-NP system is able to retain its drug load over at least the time needed for reaching the central nervous system.

### 3.5. DAL Digestion by CHT or PAC

**Figure 3:**
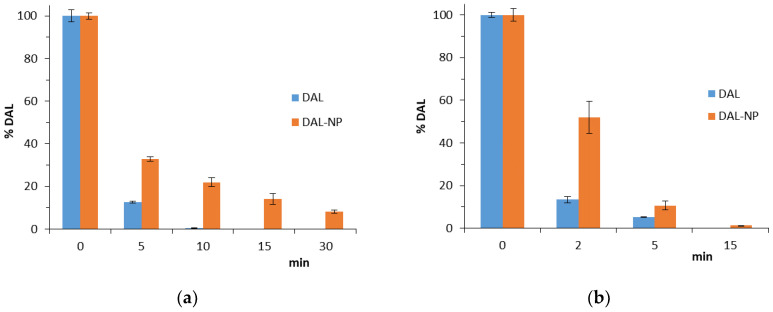
DAL and DAL-NP digestion: (**a**) starting from 5 min after addition of α-chymotrypsin (CHT); (**b**) starting from 2 min after addition of PAC. Data are the means ± standard deviation (SD) of three independent experiments.

Figure 3a shows the DAL degradation kinetics by CHT, conducted under controlled conditions simulating the digestion in intestinal environment. The DAL degradation was fast; in fact, after only 5 min, a reduction by as much as 90 ± 0.41% of substrate was observed, as shown in Figure 3a. The degradation process was slowed down when the DAL was encapsulated in NP, in which case, indeed, the substrate reduction was limited to 60 ± 0.47%.

Figure 3b shows the DAL degradation kinetics by PAC. This enzyme was seen to be much more aggressive than CHT in degrading the peptide. The free DAL was reduced by 85 ± 1.39% of initial content just 2 min after adding the enzyme, and no peptide was present after 5 min. More resistant to degradation was the peptide encapsulated in NP (about 47.5% reduction in 2 min and 100% reduction in 15 min).

### 3.6. Cell Studies

#### 3.6.1. Cytotoxicity Studies of Caco-2 and bEnd.3

**Figure 4:**
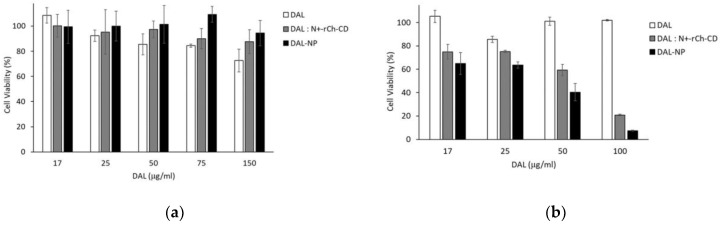
Cytotoxicity studies with (**a**) Caco-2 cell line, and (**b**) bEnd.3 cell line. The samples tested had equal DAL content, as free DAL (DAL), or DAL complexed with polymer (DAL/N^+^-rCh-CD), or as nanoparticles (DAL-NP). Data are the means ± standard deviation (SD) of three independent experiments.

The results of the cytotoxicity studies with Caco-2 by the WST-1 assay are reported in Figure 4a. It was observed that the samples tested had a low cytotoxicity at all concentrations. In particular, in terms of DAL/N^+^-rCh-CD, the cell vitality was maintained at values >80%, even at the highest DAL concentration used (150 µg/mL). The NP showed the same behavior as the non-aggregated polymer. On the other hand, with free DAL, a toxicity increase with increasing concentration was observed, although the vital cell percentage was still >70%.

The results of the cytotoxicity studies with bEnd.3 are shown in Figure 4b. It is seen that the murine endothelioma cell line was much more sensitive than Caco-2. Concerning the samples brought into contact with DAL, DAL/N^+^-rCh-CD complex, or DAL-NP, the assay evidenced a trend to reduce the vitality, which showed very low values, about 10%, with the highest concentration tested of DAL/N^+^-rCh-CD complex and DAL-NP, corresponding to 100 µg/mL DAL.

#### 3.6.2. Studies of DAL Permeation across Cell Monolayers

Following cytotoxicity evaluation of the systems under study, drug permeation studies across cell monolayers, apt to assess the permeation of DAL either free or encapsulated in NP, were carried out. Caco-2 and bEnd.3 monolayers were chosen to simulate the intestinal epithelium and the BBB, respectively. FITC labeled DAL-NP was used to concurrently monitor monolayer crossing by vehicle (NP), DAL disappearance from the apical chamber, and DAL appearance in the basolateral chamber.

#### 3.6.3. Permeation across Caco-2 Monolayers

The study was carried out with the Caco-2-differentiated cell monolayer after 24 days from seeding. After such an incubation time, the cells, grown up on the porous supports of the Transwell plates, polarized and differentiated, forming a monolayer with apical functional tight junctions and microvilli, forming brush border [23]. The maintenance of the monolayer integrity all across the study was monitored by measuring the transepithelial electric resistance (TEER). The presence of either the DAL-NP or the free DAL caused a TEER reduction to about 75% of the initial value. This effect was reversible; indeed, after 24 h from the start of the experiment, the monolayer resumed the initial TEER value, as shown in Figure S3.

The free DAL sample was readily degraded in the apical chamber by the enzyme activity of the monolayer cells, and thus only 8.67 ± 0.80% of the drug was still present at the end of the experiment (3 h) [15]. The peptide degradation was such that its presence in the basolateral chamber could never be evidenced, in agreement with previous studies [15].

**Figure 5:**
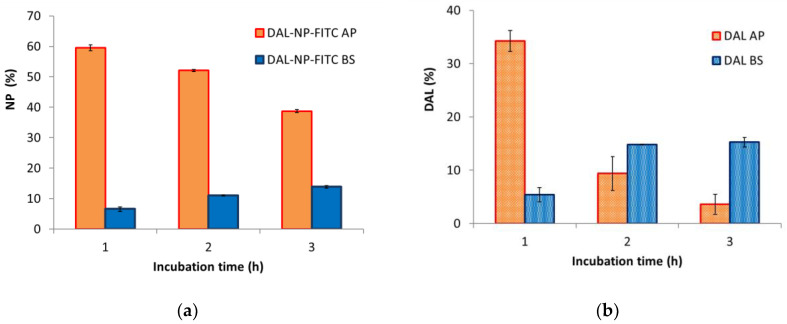
Permeation through Caco-2 monolayers. Time course of content in apical (AP) and basolateral (BS) chambers of (**a**) polymeric carrier DAL-NP-fluorescein isothiocyanate-dextran (FITC), made fluorescent with covalently labelled FITC, and (**b**) drug carried by the nanoparticles (DAL); data are the means ± standard deviation (SD) of three independent experiments.

In Figure 5, the data for the DAL-NP permeation experiment are reported. The data were obtained by monitoring either the FITC-labeled vehicle DAL-NP (Figure 5a) or the carried drug DAL (Figure 5b) in both the apical and basolateral chambers. The nanoparticle percentage in the apical chamber turned out to be decreasing in time, whereas an opposite trend was observed in the basolateral chamber, thus confirming a carrier permeation at longer times (Figure 5a). It can also be noted in Figure 5b that DAL was measurable in the basolateral chamber, and that its concentration was increasing over time; concurrently, it was decreasing in the apical chamber, mostly in the 1–2 h interval.

#### 3.6.4. Permeation across bEnd.3 Monolayers

The brain murine endothelioma bEnd.3 cell line (ATCC-CRL-2299) has been used as a model system for the permeation studies across the BBB [24,25]. Although this model has a lower TEER than the BBB, it still appeared to be suitable for the objective of the present study. In fact, it is easy to predict that the NP permeation occurs through a biological mechanism known to be unaffected by TEER. Furthermore, according to a recent study [26], bEnd.3 was found to have a higher lysosomal degradation activity than primary cells, therefore making it particularly suitable for studies involving biological permeation mechanisms such as transcytosis. For this reason, this model has recently been widely used to study the permeability of nanosystems through the BBB [27,28,29]. The paracellular pathway permeability to fluorescein (Figure S4), the monolayer integrity, and adequate cell differentiation by the hematoxylin/eosin coloring and the fluorescence analysis (scanning laser confocal microscopy) (Figure S5) were verified. Images show evidence of the formation of a homogeneous monolayer with well-marked and distributed tight junctions. However, the immunofluorescence images show, as previously reported [24], that the occludin was scattered between the cytoplasm and the cell membrane. The monolayer had a 100% TEER (corresponding to 180 Ω cm^2^), a value that was maintained all along the permeation experiment with both the DAL and DAL-NP samples (Figure S6).

The free DAL sample, in the absence of the vehicle, in the basolateral chamber was below the analytical limit, indicating that the peptide alone was unable to cross the monolayer while the peptide percentage in the apical chamber kept decreasing over time, probably due to degradation (Figure S7).

**Figure 6:**
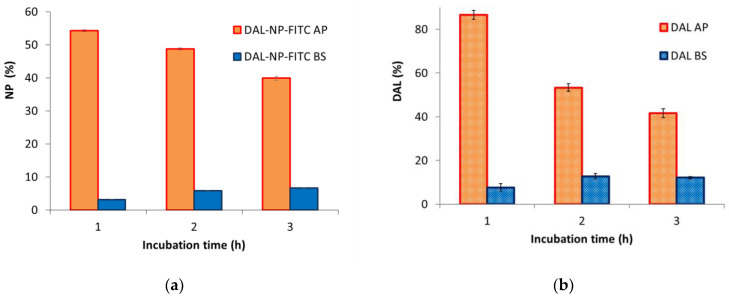
Permeation through bEnd.3 monolayers. Time course of content in apical (AP) and basolateral (BS) chambers of (**a**) polymeric carrier DAL-NP-FITC, made fluorescent with covalently labelled FITC, and (**b**) drug carried by the nanoparticles (DAL); data are the means ± standard deviation (SD) of three independent experiments.

Figure 6 shows permeation profiles for FITC-labeled vehicle DAL-NP (Figure 6a) and carried drug, DAL, in apical and basolateral chambers (Figure 6b).

In the case of NP, the fluorescence in the apical chamber kept decreasing while that in the basolateral chamber kept increasing over the 3 h of the experiment (Figure 6a). A similar trend was observed for the drug, meaning that DAL permeated thanks to the NP vehicle, showing an increasing trend in the first 2 h of the test, and remaining unvaried between the second and the third hour (Figure 6b). In the apical chamber, the DAL content decreased in time in a progressive, slower way than it did with the Caco-2 monolayer. Indeed, about 90% and 41% values were found after 1 and 3 h, respectively, i.e., much higher percentages than those found in the apical chamber with the Caco-2 cell line at equal times.

**Figure 7:**
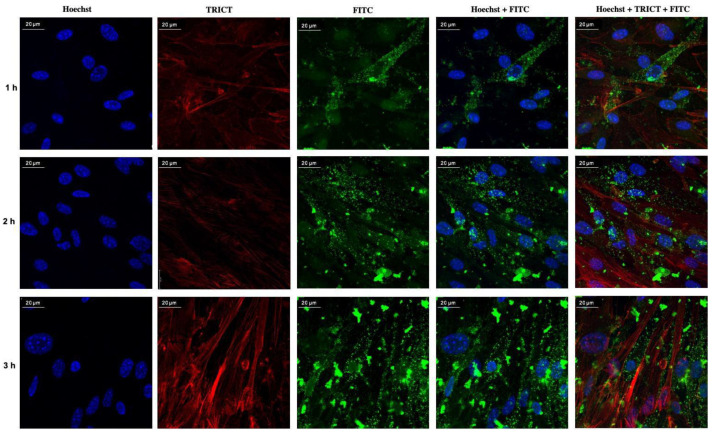
Confocal fluorescence micrograph panel for bEnd.3 monolayers used for permeation studies with FITC-labeled DAL-NP at incubation times of 1, 2, and 3h. Single acquisitions of channels: blue for Hoechst-labeled cell nuclei, red for Tetramethylrhodamine-labeled actin filaments (TRICT), green for the internalized NP (ImageJ software, National Institutes of Health, Bethesda, Maryland, USA, 80×).

The confocal fluorescence micrographs of the monolayers as isolated at each time interval of the permeation study are shown in Figure 7. A remarkable NP internalization (green) was observed with a widespread distribution in the whole cell body. Such an internalization was visible from the very first hour of experiment and lasted until the end. From this first qualitative investigation, an increased NP accumulation in cells with longer contact times resulted. Moreover, by comparing these images with a bEnd.3 control (Figure S8) not brought into contact with NP, it can be deduced that the cell monolayer and cell morphology were not altered.

## 4. Discussion

The present work has dealt with the preparation and physicochemical, enzymatic, and biological characterization of nanoparticle systems based on the semi-synthetic chitosan derivative N^+^-rCh-CD, with pendant side chains containing quaternary ammonium residues, grafted with MCD and loaded with DAL, a hexapeptide of pharmaceutical interest. In the DAL amino acid sequence, Tyr-d-Ala-Gly-Phe-Leu-Arg, two aromatic amino acids, Tyr and Phe; a long aliphatic chain amino acid, Leu; and a terminal amino acid with strong basic characteristic, Arg, were observed. Then, DAL as a whole was found to have a low molecular weight (725.85 Da), an average value of hydrophobia (0.37, based on amino acid content), and a neat charge of +2 in physiological conditions [30]. Due to its peptide nature and physico-chemical characteristics, the oral DAL bioavailability is poor. Moreover, according to the literature, the peptide is readily degraded even when administered by the intravenous route; hence, it is unable to attain the central nervous system [31]. The possibility of taking advantage of DAL-MCD complexes to increase the DAL oral bioavailability stemmed from both literature notions [16] and previous studies on N^+^-rCh-CD [15]. β-Cyclodextrin has shown a particular affinity for aromatic amino acids, such as tyrosine [32], and a behavior to slow down enzyme degradation kinetics [33]. In the present work in particular, an N^+^-rCh-CD was obtained, which contained 52% by weight of MCD and was much more soluble than the polymer conjugates obtained previously [13,14], therefore allowing a better handling.

NMR studies with model solutions containing DAL and MCD [15] in physiological conditions showed that the DAL–cyclodextrin interaction consisted in DAL inclusion within the oligosaccharide cavity and mainly involved the aromatic moieties of the tyrosine units, although interactions with phenylalanine and the alkyl residues of the leucine and alanine units were also observed. A stronger interaction of the aromatic than the aliphatic components resulted from the NMR studies.

The complexation studies indicated that, despite the possibility of interactions occurring between MCD and different amino acids of the peptide chain, the prevailing DAL-MCD stoichiometric ratio in the complex was 1:1. Moreover, the terminal Tyr residue was responsible for the interaction with the cyclodextrin cavity. The fluorescence variations due to DAL, in the case where the complex was formed with the polymer bound cyclodextrin (N^+^-rCh-CD), point to a more marked interaction and variation of the peptide chemical environment in the presence of N^+^-rCh-CD. This observation was confirmed during the assessment of the association constant. With respect to previous studies where the MCD grafting led to association constants by one order of magnitude greater than that of unbound MCD [13,14], the increase observed with this derivative was not as marked, yet significantly greater than that of MCD, with 403 M-1 vs. 126 M-1 of N^+^-rCh-CD and native MCD, respectively. In general, CD grafting to the chitosan backbone provides a positive contribution to complex formation. In this specific case, a high substitution degree was obtained, which lowered the polymer contribution compared to that of the native CD.

Interestingly a higher MCD substitution degree (52 wt%) on N^+^-rCh led to a lower toxicity degree of the resulting product to the cell lines studied. Indeed, while a cell vitality of 58% at the DAL concentration of 150 µg/mL was observed with the lowest substitution degree derivative (22 wt%) [15] the derivative studied in the present work showed about 90% vitality at the same concentration (Figure 4). This consideration is in line with what was already observed, i.e., the toxicity of the quaternary derivative was shielded by the covalently bound cyclodextrin [15]. This trend was improved by the polymer change into NP. Indeed the toxicity study on the Caco-2 line highlighted that the use of NP further reduced the toxicity of the DAL/N^+^-rCh-CD complex to a null value (vitality 100%) over the whole NP concentration interval studied.

The numerous studies carried out on chitosan-based formulations for macromolecule delivery is mainly justified by the positive charge of chitosan, which is capable of interacting with negatively charged molecules, such as DNA and some proteins, forming complexes [34]. Moreover, the chitosan NP are very good absorption enhancers for oral drug administration [35,36]. From previous studies the N^+^-rCh-CD polymer resulted in effective promotion of mucosal absorption of actives barely soluble in aqueous vehicles, such as dexamethasone [14].

The results obtained in the present work showed that the DAL-NP formulation, prepared by ionotropic gelation, has optimal characteristics from both chemical and biological viewpoints. The physico-chemical characterization evidenced a homogeneous particle size distribution, a neat positive charge, and a low polydispersity index. The formulation is also characterized by a high peptide encapsulation efficiency (about 89%), thus ensuring a very low waste of this expensive therapeutic material.

In a previous work [15], it was demonstrated that the enzymatic hydrolysis rate of DAL in the freshly prepared DAL/N^+^-rCh-CD complex was slowed down by about threefold, while neither the precursor ammonium polymer, nor the cyclodextrin, nor their mix showed any significant slowdown of DAL hydrolysis processes. Following these results, the DAL-NP were subjected to digestion by CHT or PAC studies, which confirmed that the polymeric nanostructures can increase the peptide stability against enzymatic degradation, also in this case prolonging the hydrolysis times by at least three times (Figure 3). However, the NP did not show any more ability to protect DAL from degradation than the complex DAL/N^+^-rCh-CD. This could have been due to using DAL-NP not purified from non-encapsulated DAL (about 11%).

The in vitro DAL-NP permeation studies were carried out with Caco-2, which are widely used to simulate the intestinal environment in studies of oral formulations, and the bEnd.3 brain murine endothelioma cell line to simulate the BBB [24,25]. Toxic effects of formulations were determined to select the highest peptide/carrier concentration tolerated for the permeation tests (Figure 4). As it was reported, the Leu-enkephalin transport across Caco-2 monolayers corresponds to zero value of apparent permeability [37]. This value, in line with the data in Figure 3, was related to a rapid digestion by the intestinal peptidase [38,39,40]. The sample of DAL alone was readily degraded in the apical chamber by the enzymatic activity of the monolayer cells, which was the main responsible for a null permeation, such that it could not be determined in the basolateral chamber. On the other hand, with the DAL-NP-FITC it was possible to collect the DAL kinetic data in the basolateral chamber. This demonstrates that the DAL-NP not only protected DAL from enzymatic degradation, but also promoted DAL absorption (Figure 5). In our previous study, we found that the soluble macromolecular complex DAL/N^+^-rCh-CD was able to protect DAL from enzymatic degradation, but it was not able to promote its absorption through the Caco-2. Indeed, DAL transport through Caco-2 is mainly of the transcellular type, since the paracellular permeation via tight junctions (hydrodynamic radius RH ≈ 2 nm) is closed to DAL-NP; in fact, it is only open to small solutes or macromolecules with RH in the 2–4 nm range (corresponding to an Mv ≈ 30 Kg/mol) and is bound to chain flexibility and conformation [41,42]. A transient reduction of the TEER value was observed in this study, which suggests that the NP maintained the ability to interact with the tight junctions, typical of the quaternary ammonium derivatives of chitosan [43,44,45].

Concerning the bEnd.3 cell line, the cytotoxicity tests showed a greater sensitivity, at the different concentrations tested, than Caco-2. Moreover, greater percentages of residual DAL were found in the apical chamber, at equal incubation times, with respect to Caco-2. This indicates that the Caco-2 cells are endowed with a much richer and more complex enzyme system, and hence are much more effective in degrading the peptide than the bEnd.3, despite the much greater sensitivity of the latter resulting from the cytotoxicity tests. The hypothesis of DAL permeating via the transcellular route in the bEnd.3 case is supported by the photographic material obtained by confocal microscopy, wherein the FITC labeled DAL-NP internalization within cells is evident (Figure 7).

## 5. Conclusions

The DAL-NP presented in this study as a system for transport of orally administered DAL to the central nervous system showed the ability to protect DAL from enzymatic degradation, even if not to a greater extent than the soluble macromolecular complex DAL/N^+^-rCh-CD. Nevertheless, unlike the complex, the DAL-NP also promoted DAL absorption across monolayers of either Caco-2 or bEnd.3 by the transcellular route, probably by a biological internalization mechanism. DAL-NP was found to be able to slow down DAL release at least for the time necessary for the nanosystem to be absorbed and attain the central nervous system. The kinetic data showed that the DAL degradation in simulated digestive fluids was much faster than that observed with the intestinal cell model. Moreover, since the DAL-NP absorption takes place mainly in the intestine, the NP could be inserted in gastro-resistant capsules in order to avoid their eventual gastrointestinal GI degradation and the unfruitful release of DAL in the stomach. It can be concluded that, although the results obtained in the present work are very encouraging, in vivo experiments are needed to state that the DAL-NP system is actually able to transport DAL administered per os to the central nervous system. However, in the present paper, for the first time, an innovative delivery system was developed with a polymer the synthesis of which had already been tried but with different results. The DAL-NP were shown to be able to cross the monolayer of either Caco-2 or bEnd.3, carrying significant DAL amounts.

## Data Availability

Data is contained within the article or supplementary material.

## Supplementary Materials

The following are available online at https://www.mdpi.com/1999-4923/13/1/5/s1: Figure S1: Job’s plot for the DAL/Me-β-CD for the 0, 1 h, and 24 h times. Figure S2: Job’s plot for the DAL/N^+^-rCh-CD for the 0, 1 h, and 24 h times. Figure S3: Transepithelial electric resistance (TEER) of Caco-2 monolayer as a function of time in the presence of DAL in solution (DAL) or in the particulate form (DAL-NP). Figure S4: Fluorescein (NaF) amount permeated vs. time through bEnd.3 at two different initial concentrations (50 and 100 µg/mL). Figure S5: Micrographs of the differentiated bEnd.3 monolayer after 10 days culture. Cells labeled with (a) hematoxylin/eosin (clear field microscopy, 10×), (b) micrography of monolayer with single channel of green for occludin antibody, (c) micrography of monolayer with single channel of red for anti-ZO-1 antibody, and (d) micrograph with merge of red (anti-ZO-1) and blue for labeling of nuclei (HOEST). Images elaborated with the ImageJ software (confocal scanning laser microscopy, 20×). Figure S6: Transepithelial electric resistance (TEER) of bEnd.3 monolayer as a function of time in the presence of DAL in solution (DAL) or in the particulate form (DAL-NP). Figure S7: Permeation studies of free-DAL solution across bEnd.3 monolayers. Variation of DAL concentration in the apical chamber (AP); DAL presence in the basolateral chamber was not detectable. Figure S8: Time course of DAL content in apical (AP) chamber of bEnd.3 monolayers. Figure S8: Control of the bEnd.3 monolayer used for the permeation studies with fluorescein-labeled nanoparticle samples. Acquisition of single channels (a) blue: Hoechst-labeled cell nuclei; (b) red: TRCT-labeled actin filaments; (c) merge acquisitions elaborated with the ImageJ software (confocal scanning laser microscopy, 20×).
