# Supplementary material for: Nanoparticles Based on Quaternary Ammonium Chitosan-methyl-β-cyclodextrin Conjugate for the Neuropeptide Dalargin Delivery to the Central Nervous System: An In Vitro Study
Source: Pharmaceutics. 2020 Dec 22;13(1):5. doi: 10.3390/pharmaceutics13010005 (PMC7822029; doi:10.3390/pharmaceutics13010005)
Supplement: Supplementary file 1 [file pharmaceutics-13-00005-s001.pdf]

*pharmaceutics*

MDPI

# Supplementary Materials: Nanoparticles Based on Quaternary Ammonium Chitosan-methyl-β-cyclodextrin Conjugate for the Neuropeptide Dalargin Delivery to the Central Nervous System: An In Vitro Study

**Chiara Migone [1], Letizia Mattii [2,3], Martina Giannasi [1], Stefania Moscato [2,3], Andrea Cesari [4], Ylenia Zambito [1,3,*] and Anna Maria Piras [1]**

**Citation:** Migone, C.; Mattii, L.; Giannasi, M.; Moscato, S.; Cesari, A.; Zambito, Y.; Piras, A.M. Nanoparticles Based on Quaternary Ammonium Chitosan-methyl-β-cyclodextrin Conjugate for the Neuropeptide Dalargin Delivery to the Central Nervous System: An In Vitro Study. *Pharmaceutics* **2021**, *13*, 5. https://doi.org/10.3390/pharmaceutics13010005

Received: 12 November 2020
Accepted: 19 December 2020
Published: 22 December 2020

**Publisher's Note:** MDPI stays neutral with regard to jurisdictional claims in published maps and institutional affiliations.

**Copyright:** © 2020 by the author. Licensee MDPI, Basel, Switzerland. This article is an open access article distributed under the terms and conditions of the Creative Commons Attribution (CC BY) license (http://creativecommons.org/licenses/by/4.0/).

## 1. Materials and methods

### *1.1. Immunofluorescence studies*

Cells of the b.End.3 with 5x10$^4$ cells per well concentration were seeded on 12 well Transwell™ plates on polyester 0.4 μm pore filters and incubated 10 days at 37 °C in an atmosphere containing 5%$CO_2$. Full medium (1.5 ml) was added to the basal chamber, while a 0.5 ml volume (including cells) was added to the apical chamber. The medium was renewed every 2 days. After 6 days of culture the medium of the basolateral chamber was replaced by a serum-free medium and cultured 4 more days before the experiments.

To gain information concerning the actual formation of the cell monolayer simulating the blood-brain barrier, the Zonula occludens-1 (ZO-1) and occluding were labeled. For this purpose after 10 days of culture the bEnd.3 cells grown on the Transwell™ filter were washed 3 times with cold PBSA to proceed to a pre-fixation, subsequently they were fixed with 1 ml of 3.7% paraformaldehyde in PBS 1X for 10 min. Three 5 min washings with 1 ml each of PBS, followed by permeabilization with Triton X-100 (0.2% in Normal Goat Serum, NGS, 1% in PBS) for 10 min. Cells were washed 3 times with PBS 1X and incubated 1h with 10% NGS at 4 °C under stirring. Subsequently the cells were incubated with the primary antibodies for ZO-1 and occludin, 1:250 and 1:100, respectively, in 1% NGS overnight at 4 °C. Then the cells were thoroughly washed with NGS 1% and incubated 2 h in the dark with the corresponding fluorescent secondary antibody, diluted 1:500 in NGS 1% (Alexa Fluor 568 anti-rabbit for ZO-1 Alexa Fluor 488 anti-mouse for occludin). Thereafter the cells were fixed and the Transwell™ filters were gently detached and placed on microscope slides to which two drops containing the dye for nuclei (Hoechst 33342) were added. The samples were observed on a laser scanning confocal microscope using the excitation wavelengths of 568, 488 and 350 nm for the ZO-1, occludin and the nuclei, respectively.

### *1.2. Hematoxylin/eosin (E/E) coloration*

The coloration was carried out after 10 days culture by the following protocol: the filters were washed 5 min with deionized water. For coloration of nuclei hematoxylin was added for 5 min, subsequently the filters were washed with deionized water for an equal time and incubated 5 min with eosin for cytoplasm coloring. After treatment, the filters were observed under an inverted optical microscope.

### *1.3. Sodium fluorescein (NaF) permeation studies through bEnd.3 cells*

These studies, carried out to verify the permeability of molecules that pass through the paracellular path across the bEnd.3 monolayers, were performed as described in paragraph 2.7.3. The initial NaF concentration applied in the apical chamber was 50 or 100 μg/mL.

## 2. Results

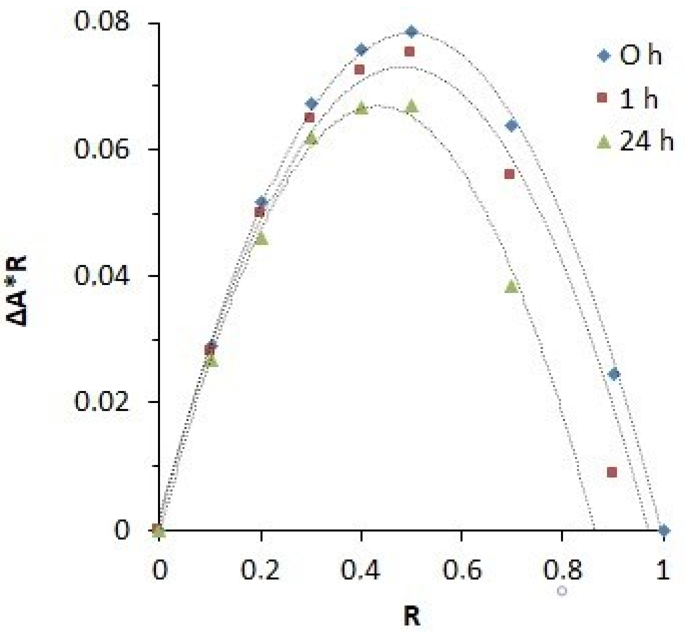


**Figure S1.** Job's plot for the DAL:Me-β-CD for the 0, 1 h, 24 h times.

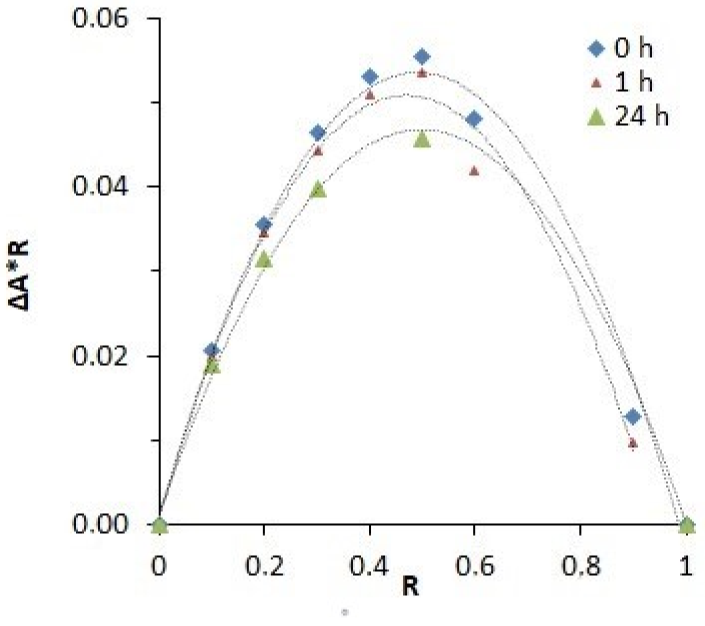


**Figure S2.** Job's plot for the DAL: $N^+$-rCh-CD for the 0, 1 h, 24 h times.

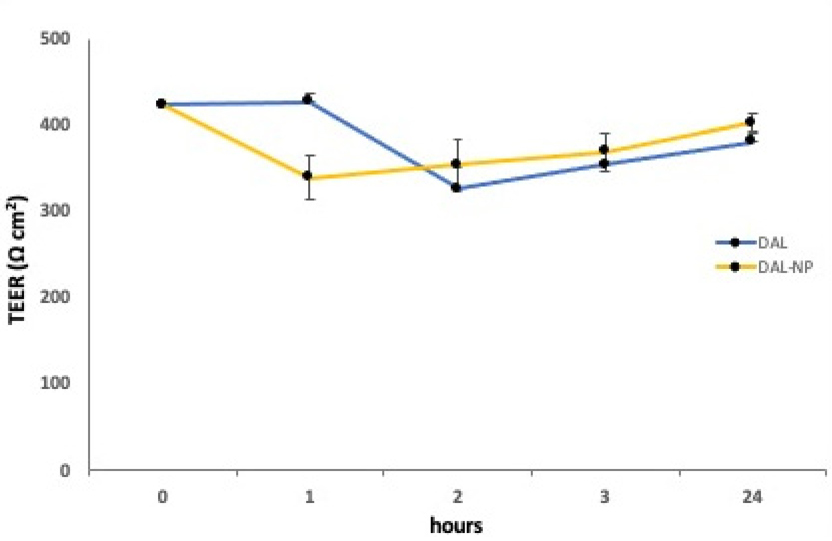


**Figure S3.** Transepithelial electric resistance (TEER) of Caco-2 monolayer as a function of time in the presence of DAL in solution (DAL) or in the particulate form (DAL-NP).

The permeation test was carried out in order to study the apparent permeability (Papp) of bEnd.3 monolayers to NaF permeation through the paracellular pathway. The plot obtained is shown below (Fig. S5).

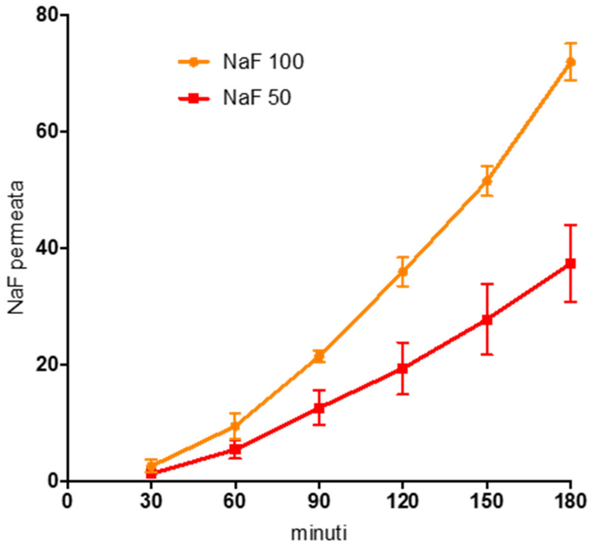


**Figure S4.** Fluorescein (NaF) amount permeated vs time through bEnd.3 at two different initial concentrations (50 and 100 μg/mL).

The slope of the linear portion of the straight line relative to the plot (Figure S5) allowed to calculate the $P_{app}$, using the equation:

$P_{app}$= dM/dt 1/(A$C_0$)

where dM/dt 1/A is the plot slope and $C_0$ is the initial NaF concentration used in the permeation experiment.

For the two samples with different initial NaF concentrations, the following values of $P_{app}$ were found: NaF (50 μg/mL) = 0.51 $10^{-6}$ cm/sec and $P_{app}$; NaF (100 μg/mL) = 0.52 $10^{-6}$ cm/sec, which confirm the cells are confluent and permeability does not depend on the NaF concentration applied.

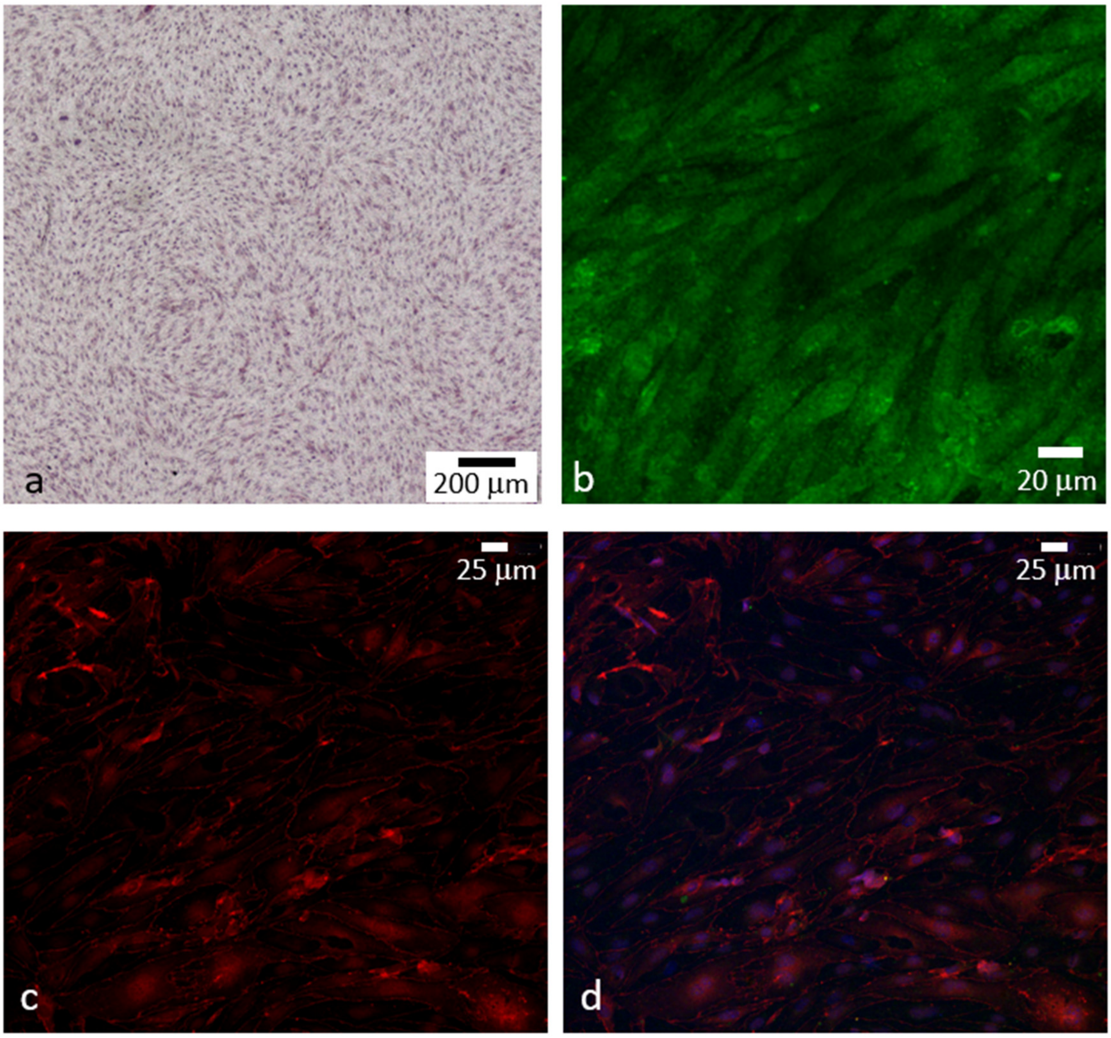


**Figure S5.** Micrographs of the differentiated bEnd.3 monolayer after 10 days culture. Cells labeled with a) hematoxylin/eosin (clear field microscopy, 10X); b) micrography of monolayer with single channel of green for occludin antibody; c) micrography of monolayer with single channel of red Figure 1. antibody; d) micrograph with merge of red (anti-ZO-1) and blue for labeling of nuclei (HOEST). Images elaborated with the Image J software (confocal scanning laser microscopy, 20×).

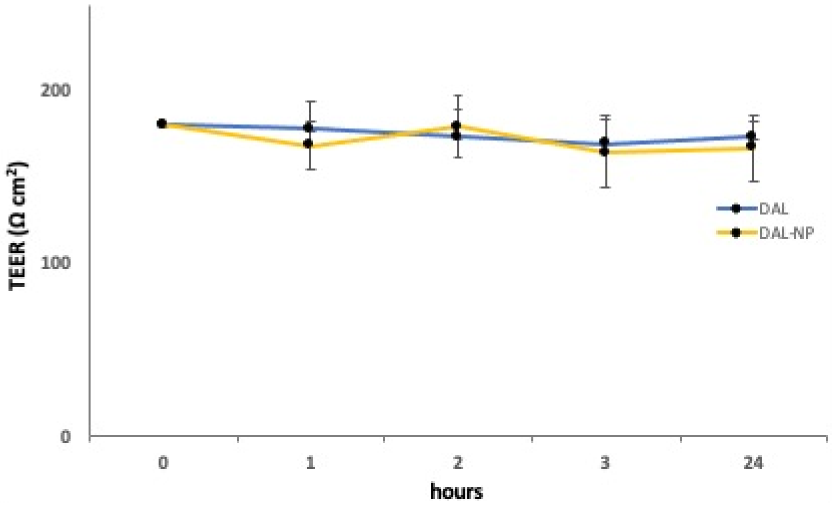


**Figure S6.** Transepithelial electric resistance (TEER) of bEnd.3 monolayer as a function of time in the presence of DAL in solution (DAL) or in the particulate form (DAL-NP).

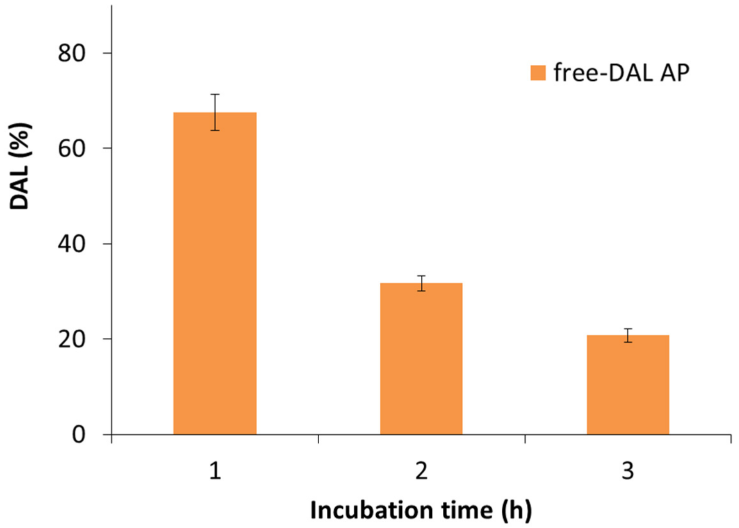


**Figure S7.** Permeation studies of free-DAL solution across bEnd.3 monolayers. Variation of DAL concentration in the apical chamber (AP); DAL presence in the basolateral chamber was not detectable.

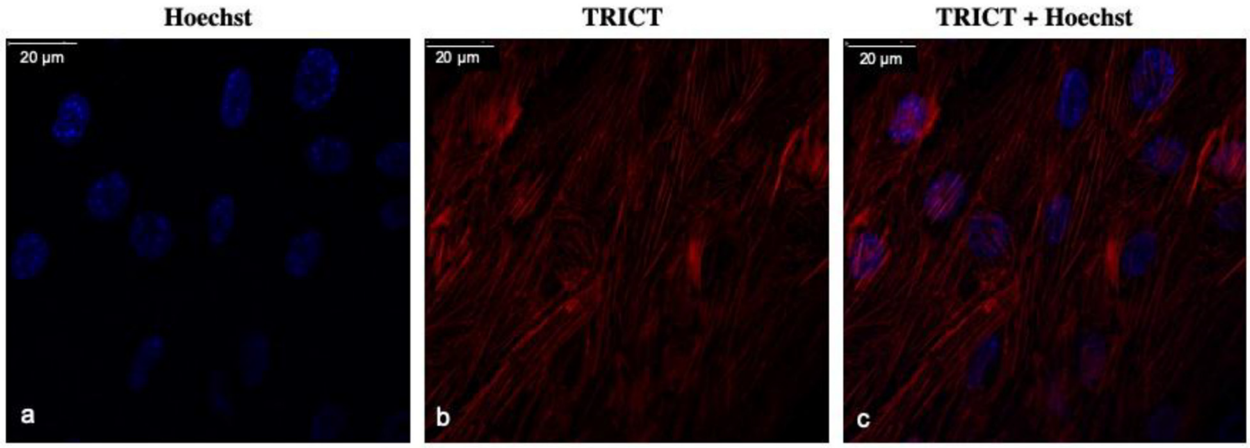


**Figure S8.** Control of the bEnd.3 monolayer used for the permeation studies with fluorescein labeled nanoparticle samples. Acquisition of single channels (**a**) blue: Hoechst labeled cell nuclei; (**b**) red: TRCT labeled actin filaments; (**c**) merge acquisitions elaborated with the Image J software (confocal scanning laser microscopy, 20×).
